# The Role of Community Engagement in Successful Recruitment of Research Participants During the COVID-19 Pandemic

**DOI:** 10.1089/heq.2024.0003

**Published:** 2024-05-15

**Authors:** Kyra Oziel, Jessica D. Hanson, Karen Little Wounded, Serea Darnell, Dedra Buchwald

**Affiliations:** ^1^ Institute for Research and Education to Advance Community Health, Washington State University, Seattle, Washington, USA.; ^2^ Department of Applied Human Sciences, University of Minnesota Duluth, Duluth, Minnesota, USA.; ^3^ Missouri Breaks Industries Research Inc., Eagle Butte, South Dakota, USA.

**Keywords:** community-based participatory research, COVID-19, recruitment, randomized controlled trials, American Indian tribes

## Abstract

**Background:**

Our research team was in the process of recruiting American Indian and Alaska Native (AIAN) women for a community-based intervention to prevent alcohol-exposed pregnancy when the COVID-19 pandemic began. Safety measures adopted at the tribal, state, and national level required us to rethink and revise study protocols. We followed the principles of community-based participatory research, especially community engagement. The goal of this article is to report the recommendations from local AIAN field staff and the community advisory board that enabled us to exceed our prepandemic recruitment goal.

**Methods:**

First, we developed a list of major adaptations and mapped each one onto our recruitment timeline to assess its effect on subsequent enrollment. Second, we surveyed the two AIAN field staff who led recruitment and an administrative staffer at the study site and conducted a qualitative analysis of their responses.

**Results:**

Our revised project timeline presents the major adaptations that led to our successful recruitment, as verified by qualitative data from field staff. These adaptations included expanding our social media presence, expanding recruitment to a nearby urban site, implementing a “refer a friend” program, and recruiting through local media outlets. Most important was having local AIAN staff who cultivated a nonjudgmental space for potential participants to talk about sensitive topics.

**Discussion:**

We not only met our prepandemic recruitment goal but exceeded it by 16.6%. The input of our community advisory board and the efforts of community-based staff were essential in achieving success during the unprecedented conditions of the COVID-19 pandemic.

## Background

Health research with American Indian and Alaska Native (AIAN) people has been complicated by the legacy of European colonization and the resulting historical trauma experienced by Indigenous Americans.^1^ Negative perceptions of health research in AIAN communities have been further exacerbated by past studies conducted with insufficient ethical protections or none at all.^2–4^ A growing literature supports the efficacy of applying principles of community-based participatory research (CBPR) to academic research with AIAN populations.^5^ CBPR is a systematic effort to “incorporate community participation and decision making” that involves community partners in all stages of the research process, from conceptualization to implementation to evaluation; it often includes capacity building in study communities.^6–7^

Applying CBPR principles has been particularly important during the COVID-19 pandemic. Many AIAN communities have been disproportionately affected by the pandemic,^8^ and many tribal nations imposed strict regulations to prevent the spread of disease.^9^ Our group successfully implemented Native CHOICES, a prevention study, in tribal communities that employed a broad range of strategies to reduce the spread of COVID-19, including checkpoints on vehicular traffic, residential curfews, reductions in community services, and targeted stay-at-home orders.^9^ The principles of CBPR, as outlined by Israel et al.,^10^ that will be most prominently featured in this study, are the recognition of the community as a unit of identity, an effort to build on the strengths and resources already present within the community, a collaborative partnership between the university, the research organization, the Native community in all phases of the research, and a cyclical and incremental process throughout the changes implemented in the study.

Native CHOICES was a randomized controlled trial of the culturally adapted CHOICES intervention, focused on preventing alcohol-exposed pregnancy in preconception women. Full details are available in a separate publication.^11^ The study involved a research collaboration between Washington State University and our community partner, a small Native-owned research organization with a long history of working with AIAN communities and promoting AIAN health. The local Native-owned research organization has strong local ties and has been working in the region for over 40 years. The study was conducted at two sites in a single state in the Great Plains region of the United States: a rural reservation-based community and an urban area.

We recruited AIAN women of age 18–44 years who were at risk of alcohol-exposed pregnancy. They were randomized either to an intervention that included motivational interviewing or to a waitlist control condition with services as usual.^12^ The waitlist control design was requested by the tribal entity to allow participants who were not randomized into the intervention condition the option to receive the motivational interviewing intervention after their 6-month participation in the study. Before the pandemic, recruitment and intervention activities were conducted in person by the field staff of our community research partner, all of whom are trained Native community members with personal knowledge of the local context. When the pandemic started, our research partner chose to enforce strict protocols to protect its employees and all research participants, beyond those mandated by the state or participating tribal communities. Among them was a prohibition on public access to their offices. As a result, our recruitment stalled in March 2020. Nonetheless, ongoing engagement with local field staff and input and oversight of the team’s decisions by our study’s community advisory board ultimately enabled us to exceed our original recruitment goal of 350 participants. The community advisory board for this study consisted of community members who live on the surrounding tribal lands; they were selected through the Native-owned research organization’s longstanding ties to the community, and they were compensated for their time and input. At least 4 advisory board members would meet in a hybrid in-person and remote setting to discuss the ideas put forward for the projects running at the community partner organization on an annual and ad-hoc basis throughout the duration of the study. Field staff on this study would either attend the board meetings or receive meeting minutes for the discussed items and conveyed the perspectives of the community advisory board members back to the study team. We provide a fuller discussion of our adaptations in a separate publication.^9^ The goal of the present article is to use a project timeline and qualitative data from field staff to highlight the essential role of community engagement in successful study recruitment during the COVID-19 pandemic.

## Methods

Our study team closely monitored recruitment, which was on target to meet our original goal before pandemic restrictions began in March 2020. Thereafter, we relied heavily on AIAN field staff for real-time understanding of any local perspectives, safety precautions, or tribal regulations that might affect recruitment efforts. The partnership between Washington State University and the Native-owned research organization builds upon a pre-existing decade-long relationship across dozens of prior grants in which the local expertise of staff has been repeatedly demonstrated and trust in the academic university has been earned through respectful deference to tribal sovereignty. In virtual project meetings occurring every other week, field staff, informed by personal experience and input from our project’s community advisory board, led discussions on how best to adapt our research protocols during every stage of tribal, state, and national response to the pandemic. Although bidirectional feedback was incorporated through each phase of the study, the research team felt that the resulting adaptations during the COVID-19 pandemic were invaluable to recruitment efforts, so we decided on a two-pronged approach to collect data that would verify the effectiveness of our revised approach.

First, to understand the impact of the adaptations on recruitment, two authors (K.O. and J.H.) created a list of major activities and decisions during active recruitment that they believed had an effect on achieving recruitment goals. Then they met with two other authors (K.L.W. and S.D.), both of whom worked as field staff, to ensure that the list included all relevant points.

Second, to understand the impact of the adaptations from the perspective of field staff who conducted recruitment, one author (J.H.) created a set of four open-ended questions to distribute:

1. How do you think the COVID-19 pandemic impacted research studies in general?
2. How do you think the COVID-19 pandemic impacted the Native CHOICES recruitment and retention process?
3. What were the top three things the Native CHOICES project did to recruit and retain participants during the COVID-19 pandemic?
4. What would you say was your biggest success during the COVID-19 pandemic, specific to the Native CHOICES project?

These questions were emailed to both of the members of our research partner’s field staff who conducted recruitment and intervention activities as well as an additional staff member at the research partner who was appropriately positioned to speak on the effects of the pandemic across the center’s other grants. All three provided written responses, and then the academic staff met with the two field staff members over Zoom to discuss their responses and provide an opportunity for them to expand on their perceptions. Academic staff took notes on this discussion.

The first author (K.O.) then aggregated the responses and performed a conventional content analysis to identify themes.^13^ Two other authors who were also field staff (K.L.W. and S.D.) then joined this phase of analysis to verify themes and review our existing list of actions. This approach led to a revised list, whose components were mapped onto our recruitment tracking timeline to assess the timing and subsequent influence of each action on study recruitment.

## Results

**FIG. 1.**
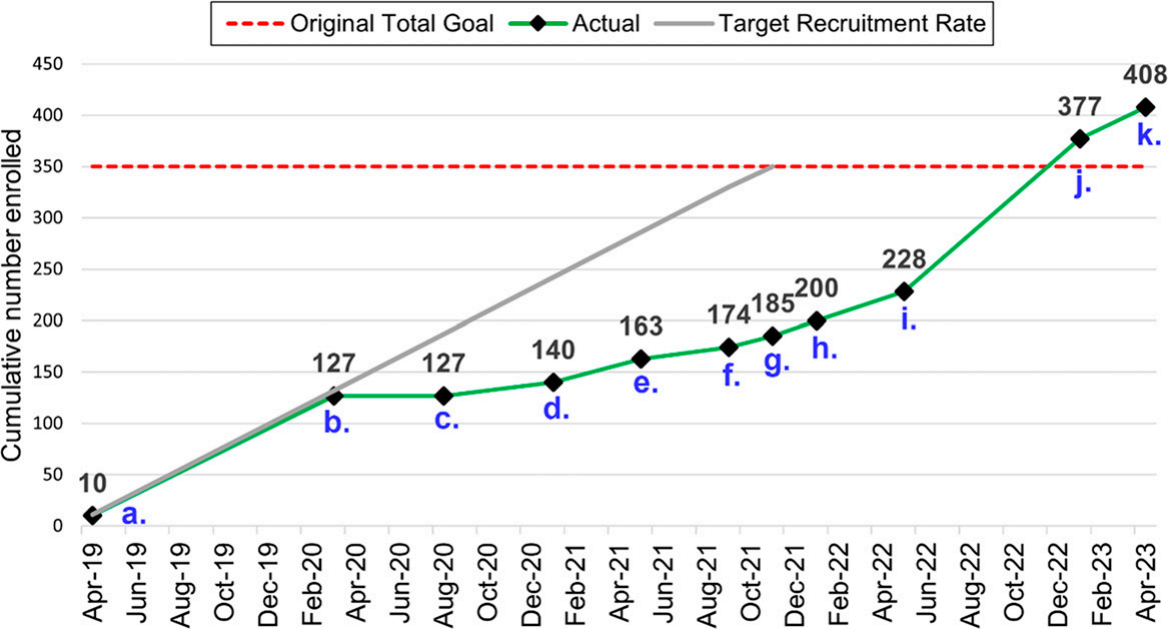
Timeline of Native CHOICES participant recruitment. Figure 1 shows a timeline of actual participant recruitment in green along with the initial ideal target recruitment rate in gray to the original target recruitment goal of 350 participants indicated by the dotted red line. The actual participant recruitment line also includes letters indicating the timing of key events and adaptations throughout the course of the study (a–k) as determined by the study team and confirmed by field staff qualitative data. Active recruitment began in April 2019 (a), COVID shutdowns began to take effect in March 2020 bringing the study to a halt (b), the team used feedback from our community partners to guide the decision to switch to enrollment and data collection by telephone to best meet the needs of the rural reservation-based community in August 2020 (c). After the switch to remote enrollment, staff focused additional efforts on advertising through the study’s Facebook page in January 2021 (d). In May 2021 an additional urban intervention site within the same state was added (e). We began offering a $10 additional incentive to participants enrolled in the study who referred another individual who was eligible and subsequently enrolled in the study in September 2021 and this offer persisted through the end of enrollment (f). By November 2021 in-person enrollment and data collection was again made available, telephone and in-person options were maintained for the rest of the data collection period (g). In January 2022 we began using paid Facebook advertisements targeted to our population for the urban site (h) and in April 2022 the urban site office was moved to a more pedestrian and public transit friendly location (i). In January 2023 we reached our original recruitment goal (j) and in May 2023 we finished enrollment (k), exceeding our original goal by 16.6%.

Figure 1 provides an overview of the number of participants enrolled over time during active recruitment. Each time point in the figure (labeled a–k) corresponds to a specific event or decision that was determined to have an impact on recruitment success. Active recruitment began in April 2019 (point a) and COVID shutdowns pursuant to tribal recommendations and mandates began in March 2020 (point b). After extensive discussions with our community partner, we decided in August 2020 to move entirely to remote data collection by telephone, rather than Zoom, because of technology limitations across the region (point c). This change likely encouraged recruitment and improved participant retention.

To alert participants to changes in the program and conduct additional recruitment efforts, field staff dedicated more time per week to recruiting on social media through the Native CHOICES Facebook page (point d). Field staff identified that of the existing social media platforms, Facebook was commonly used throughout the community. An urban intervention site with a large pool of eligible participants was added in May 2021 to augment recruitment (point e). In September 2021, we added a $10 incentive to any current participant who referred an eligible participant who subsequently enrolled in the study and this offer was maintained for the rest of the open enrollment period (“refer a friend,” point f). In November 2021, we reinstated our option for in-person study visits (point g), and in January 2022 the local study team began paying Facebook to boost social media posts referring to the urban site (point h). These boosted posts helped our study promotion materials on Facebook reach women who fit the age eligibility criteria within a 50-mile radius of the urban site. In April 2022, the study team moved the urban office to a new location that was more accessible to pedestrians and public transit than the original location in response to feedback from the community (point i). We reached our original recruitment goal of 350 (point j) only one month later than the date we set before the pandemic began. We then exceeded that goal by 16.6%, for a total enrollment of 408 participants in May 2023, the final month of recruitment (point k).

In addition to these metrics, input from field staff helped us understand which tactics were likely responsible for enhancing recruitment. As one field staff member stated, “The pandemic forced not only our team but all researchers working with the tribe to rethink and create new recruitment methods.” Nonetheless, field staff also noted the effectiveness of the strategies in our original, prepandemic study plan. In general, they felt that one of the most successful adaptations in our study protocols was the decision to meet participants “where they were at” in terms of flexibility, especially by adding telephone-based sessions.

Given the focus of our intervention study—preventing alcohol-exposed pregnancy—employing local field staff who cultivated a nonjudgmental space for participants to talk about sensitive topics was viewed as essential for recruitment success. Participants were not only given space to discuss such topics as alcohol use and sexual activity, but were also provided with practical resources after enrolling at the baseline visit. These included hygiene products, hairbrushes, hand sanitizer, resource guides with contact information for local birth control and other medical assistance, alcohol treatment resources, and resources for pregnant women in the surrounding area. Field staff also conducted recruitment at outdoor events, such as drive-through health fairs, drive-through food giveaways, and drive-through recruitment efforts sponsored by our local research partner for other studies as well as our own.

One component of our original study design that field staff perceived as effective was our delivery method for participant incentives. We issued checks in dollar amounts instead of gift cards because many communities where participants live have few retail locations that accept gift cards as payment. With checks, funds were instantly available without restrictions on their use. This choice let participants use the funds to purchase what they needed, when they needed it. At Washington State University research incentives are most commonly issued in the form of gift cards but since our community research partners identified this as a barrier the academic Institutional Review Board approved the use of checks for this study to accommodate the unique tribal community’s preferences. Notably, staff felt that economic factors also helped to drive enrollment, as “financial strain on some families may have made them more eager to participate in a study with compensation.”

Field staff also noted the positive impact of our grassroots approach to recruitment, especially our use of local resources to advertise and recruit. For example, staff discussed the study on a local tribally-owned radio station that often disseminates news to the region, including COVID-19 updates. That became an important way to reach many people quickly and inexpensively. Another key activity identified by staff was going door to door with recruitment bags that included information about our study, recruitment materials, information about other studies that were actively recruiting, and items such as candy and pens.

Our team listened carefully to the perspectives of local community members and met our participants “where they were at” in terms of emotional vulnerability related to the study topic, physical location, information sources, potential worries about the pandemic, and preferred methods for interacting with the study team. We also provided context-appropriate financial incentives for participating in each stage of data collection, beginning at enrollment.

## Discussion

We developed innovative solutions to the problem of recruiting AIAN participants for a health intervention during the COVID-19 pandemic. Those solutions enabled us to exceed our prepandemic recruitment goals. A possible limitation of this study is that only one staff member conducted the initial content analysis on the field staff perspective data, although this analysis was subsequently reviewed and confirmed by additional staff members.

We attribute our success to a collaborative team effort and to community-based field staff, who guided the process of adapting recruitment procedures and data collection to remote delivery, with a focus on delivery by telephone. Other studies have also reported that revising research methods or shifting intervention delivery to remote modalities maintained recruitment at functional levels during the pandemic, yet these studies also found that engagement of AIAN participants ultimately declined.^14,15^

In contrast, the adaptations implemented by the Native CHOICES team helped to maintain recruitment levels and sustain relationships with enrolled participants even during periods of enforced isolation, which were particularly challenging for remote rural residents. Among our adaptations, we highlight the “refer a friend” program; providing additional context-appropriate financial compensation for participation; conducting recruitment both in person and remotely; delivering recruitment bags containing study materials to the homes of potential participants; increasing the frequency of social media posts and targeting them to specific demographics; adding an urban recruitment site; and then moving that site to a more accessible location to facilitate the resumption of in-person visits. In addition, the qualitative data reported herein foregrounds the critical importance of employing local staff who are knowledgeable about prevailing social, regulatory, economic, and cultural conditions. Our research partner’s field staff created a supportive environment where participants felt respected and willing to engage, from baseline data collection and intervention delivery through three successive follow-up data collection visits.

Our referral incentive program was especially effective, since it enabled us to reach potential participants even when lockdown protocols limited our usual recruitment efforts. Given the personal nature of the study topic, referral from a trusted individual who was already a participant likely eased hesitation about enrolling.

## Conclusions

During the COVID-19 pandemic, most research with in-person interactions had to undergo major changes in response to the volatile health and safety landscape. This study benefited from a design that gave people in the community of interest a prominent voice in decision making. The local expertise and perspectives of AIAN field staff, who were intimately familiar with the local health care ecology and social context, were crucial factors in creating and implementing novel, effective adaptations to the original study design. After conferring with field staff, we were able to tailor our work to suit our study communities’ needs and preferences. We continued to listen to their perspectives and make further adaptations as the pandemic unfolded. Our results underscore previous findings that CBPR principles are essential for adapting to changing research environments, and that local staff are best situated to implement such adaptations. The community-engaged effort outlined in this article offers a rich and nuanced example of how to navigate the social, cultural, economic, and technological factors that influence study recruitment in all populations.

## Abbreviations Used

AIAN: American Indian and Alaska Native
CBPR: community-based participatory research
D.B.: Dedra Buchwald
IRB: Institutional Review Board
J.H.: Jessica Hanson
K.L.W: Karen Little Wounded
K.O.: Kyra Oziel
S.D.: Serea Darnell
